# Behavioral Welfare Economics

**DOI:** 10.1017/bca.2020.14

**Published:** 2020-05-07

**Authors:** Cass R. Sunstein

**Affiliations:** Harvard University, Cambridge, MA, USA, e-mail: csunstei@law.harvard.edu

**Keywords:** behavioral economics, cost-benefit analysis, heuristics, biases, D9, D91

## Abstract

A growing body of normative work explores whether and how deference to people’s choices might be reconciled with behavioral findings about human error. This work has strong implications for economic analysis of law, cost–benefit analysis, and regulatory policy. In light of behavioral findings, regulators should adopt a working presumption in favor of respect for people’s self-regarding choices, but only if those choices are adequately informed and sufficiently free from behavioral biases. The working presumption should itself be rebuttable on welfare grounds, with an understanding that the ends that people choose might make their lives go less well. For example, people might die prematurely or suffer from serious illness, and what they receive in return might not (on any plausible account of welfare) be nearly enough. The underlying reason might involve a lack of information or a behavioral bias, identifiable or not, in which case intervention can fit with the working presumption, but the real problem might involve philosophical questions about the proper understanding of welfare, and about what it means for people to have a good life.

## 1. Two claims

I offer two claims here. The first is that in light of behavioral findings, regulators should adopt a working presumption in favor of respect for people’s self-regarding choices, but only if those choices are adequately informed and sufficiently free from behavioral biases. These are important qualifications, calling for significant departures from standard economic approaches to welfare analysis.

The second is that the working presumption is itself rebuttable on welfare grounds. Even if people’s self-regarding choices involve their “direct” judgments (as defined below), regulators should not necessarily respect them. The ends that people choose might make their lives go less well. For example, they might die prematurely, or suffer from serious illness, and what they receive in return might not (on any plausible account of welfare) be nearly enough. The underlying reason might involve a lack of information or a behavioral bias, identifiable or not, in which case intervention can be made consistent with the working presumption, but the real problem might involve philosophical questions about the proper understanding of welfare, and about what it means for people to have a good life. Still, regulators should proceed with great caution, on the ground that reasonable people reasonably care about diverse goods, and they can make reasonable, and different, judgments about how much weight to give them. This point supports the working presumption and calls for humility in the face of self-regarding choices, certainly when choosers are sufficiently informed and sufficiently free from behavioral biases.

These propositions suggest that behavioral welfare economics, even as used in applied work and in government circles, must at least implicitly take a stand on the best understanding of welfare. I do not offer anything like a comprehensive account in this space, but in brief, I shall argue (i) that purely hedonic accounts ignore important aspects of what people legitimately care about; (ii) that preference-based accounts pay too little attention to behavioral biases and also to the ingredients of what is, for essentially all people, a good human life; and (iii) that objective-good accounts tend to underplay human diversity and the heterogeneous goods that matter to reasonable people. The working presumption can be seen as a pragmatic way of accommodating points (i), (ii), and (iii). With respect to both theory and practice, it aspires to an “incompletely theorized agreement” (Sunstein, 2018): a principle on which people can converge despite their disagreement or uncertainty about the most fundamental questions.

Much of behavioral economics, and much of behaviorally informed analysis of law, focuses on departures from standard accounts of rationality (see Thaler, 2015 for a clear catalogue). Exploring actual behavior, it seeks to avoid the most contentious normative questions about the relationship between choices and welfare. But an illuminating and growing body of work explores whether and in what sense economists, lawyers, and others interested in behaviorally informed public policy can continue to insist on the sovereignty of individual preferences, while also acknowledging behavioral findings (for a highly selective account, see Bernheim & Rangel, 2007; Bernheim, 2009; 2009; Allcott & Sunstein, 2015; Allcott & Kessler, 2019; Thunstrom, 2019). My central aim here is to argue for a degree of humility, captured in the working presumption in favor of respect for people’s informed and behaviorally unbiased judgments (Allcott & Sunstein, 2015).^1^ As we shall see, this presumption can be disciplined by asking a series of subsidiary questions, and it should be accompanied by a distinction between “means paternalism” and “ends paternalism,” of special relevance to behaviorally informed law and policy.

To anchor the discussion, consider the following cases:

(i) Sarah Masters recently bought a car. She considered a package with various safety featuring, including a camera designed to ensure rear visibility, but she rejected that package; it would have cost $75. Many consumers are making the same decision that Masters made.
(ii) Jerry Lancaster is overweight (and as a result, at heightened risk of getting diabetes). He would like to diet, but he is not sure how. He goes to restaurants every day for lunch, and he tends to order high-calorie items. He is not aware of the caloric content of his orders. Many consumers act as Lancaster does.
(iii) Pamela Harston is eligible for the Earned Income Tax Credit. She is aware of that fact. But, she is not quite sure how to apply. She is also very busy. She thinks that she will apply next month. She has thought that for a long time. Many people are doing as Harston does: failing to apply for benefits for which they are eligible.
(iv) Edward Ullner is in his 30s and also healthy. In the midst of a pandemic, very much like the coronavirus pandemic of 2020, he is not especially worried. He does not believe that he will fall ill, and even if he does, he does not believe that he will suffer long-term harm. He does not wear a mask, and he does not stay at home.

In all of these cases, it is at least plausible to think that the relevant agent is making some kind of mistake, one that will produce serious harm. We could also believe, certainly in the case of Ullner, that harm to others is involved. But even if we bracket that possibility, the point remains: Ullner might be endangering his own life, perhaps because of an absence of information, perhaps because of unrealistic optimism. On welfare grounds, regulators might want to respond in some way, perhaps with a nudge (Thaler & Sunstein, 2009), perhaps with an economic incentive, perhaps with a ban or a mandate (Conly, 2013).

A preview: Sections 1 and 2 explore choice-oriented methods for conducting behavioral welfare analysis. The exploration is generally sympathetic to those methods, but I shall offer some critical commentary, with reference to behavioral biases and to the contestability of any choice-oriented conception of welfare. Section 4, the heart of the article, investigates the theoretical underpinnings of choice-oriented methods. It suggests that the prominent idea of “deference to direct judgments” runs into two serious objections. The first is that the category of direct judgments is relatively small, at least for purposes of regulatory policy; we are almost always dealing with indirect judgments. The second is that the argument for respecting direct judgments depends on contentious philosophical commitments, and it is not always convincing. Section 5 identifies paths forward, with reference to the possibility of an incompletely theorized agreement on the working presumption and with specification of how to make it operational in practice.

## 2. Choices and welfare

Do people’s choices promote their welfare? For reasons that are now well known, the best answer is “sometimes.” If people lack information, they might choose poorly (Bar-Gill, 2012). If sellers exploit people’s behavioral biases, consumers’ choices may go awry (Bar-Gill, 2012; Akerlof & Shiller, 2016). Even without self-conscious exploitation of such biases, people may choose poorly because of limited attention, inertia, present bias (O’Donogue & Rabin, 2015), or unrealistic optimism (Gabaix, 2019; Sarin, 2019), potentially justifying a regulatory response (Bar-Gill, 2012; Conly, 2013; Sarin, 2019). People may make mistaken predictions about the effects of options on their welfare (Sunstein, 2019c). It is also true that people are susceptible to supposedly irrelevant factors or “frames” (Keren, 2011; Scholten *et al.*, 2019). In an opt-in system, for example, they might end up in a very different situation from where they end up in an opt-out system (Jachimowitz *et al.*, 2019). If so, what is their preference?^2^

In addition, people’s preferences may be endogenous to legal rules (Mattauch & Hepburn, 2016). Whether or not that is so, preferences and values may shift over time, and choosers may not appreciate that fact ex ante (Ullmann-Margalit, 2006; Pettigrew, 2020). People sometimes “choose for changing selves” (Ullmann-Margalit, 2006; Pettigrew, 2020). A mundane example: An enthusiastic meat-eater might come to love salad and despise meat, in part because of learning and in part because of genuine preference change. These questions raise serious challenges for efforts to base law and policy on people’s preferences, or to conduct any kind of welfare analysis on the basis of preferences.^3^ They help explain the growing effort in multiple fields to investigate subjective well-being, entirely unanchored in preferences (Kahneman *et al.*, 1997; Schkade & Kahneman, 1998; Dolan, 2014).^4^

Return in this light to the cases of Masters, Lancaster, Harston, and Ullner. Masters might suffer from a lack of information or unrealistic optimism (Sunstein, 2019b). Lancaster does not have important information, and he might also have a self-control problem. Harston appears to be a procrastinator. Ullner lacks information as well, and he might well be unrealistically optimistic. In all of these cases, some kind of regulatory intervention might increase people’s welfare. If welfare is what we care about, we will not think that regulatory abstinence is mandatory.

Some of the most careful and illuminating discussions of the underlying issues, not yet engaged in law and policy, come from by B. Douglas Bernheim (2016), who begins by noting that “standard welfare economics” associates welfare with choices. We can understand Bernheim’s work as the best and clearest effort to rescue the foundations of standard economic theory while acknowledging behavioral findings. It also bears directly on questions in policy and law, such as the legitimate domain of paternalism, and for that reason, it is worth careful attention here. Bernheim suggests that standard welfare economics invokes three general premises (Bernheim, 2016):

*Premise 1: Each of us is the best judge of our own well-being.*

*Premise 2: Our judgments are governed by coherent, stable preferences.*

*Premise 3: Our preferences guide our choices: when we choose, we seek to benefit ourselves.*

Something like these premises played a central role in the early decades of economic analysis of law (Posner, 1973), and it continues to play a significant role today.^5^ Bernheim (2016) recognizes that the resulting understanding “may fall short of a philosophical ideal,” but urges that this “should not trouble us excessively,” because it “captures important aspects of well-being and lends itself to useful implementation.” That is an eminently reasonable claim, but it leaves open questions, and it remains to be specified,. In some cases, it might also point in the wrong directions. Consider, for example, the question whether a soda tax is a good idea (Allcott *et al.*, 2019); whether energy efficiency regulations can be justified as a response to consumer mistakes (Allcott, 2016); whether heavy taxes on cigarettes might make smokers better off (Gruber & Mullainathan, 2005); whether motor vehicle safety regulation might provide people with “experience goods,” such as rear visibility, and thus improve their lives by their own lights (Sunstein, 2019b); and whether nudges or mandates might be a good idea in the context of a pandemic (to prevent harm to choosers, not to others). In these and other cases, falling short of a philosophical ideal might turn out to be a fatal flaw.

And what, exactly, is meant by the claim that the standard understanding “captures important aspects of well-being?” Perhaps the suggestion, empirical in nature, is that much of the time, satisfaction of people’s preferences does, as a matter of fact, promote their well-being (properly understood), simply because they know what they like.^6^ Bernheim (2016) makes something like this claim, invoking “the central Cartesian principle that subjective experience is inherently private and not directly observable.” He adds: “We know how we feel; others can only make educated guesses. These considerations create a strong presumption in favor of deference to our judgments.”

Bernheim (2016) also makes a separate argument, involving autonomy rather than welfare: “my views about my life are paramount because it is, after all, *my* life.” This is a Kantian idea, suggesting the importance of respect for choosers, even if they err. People are ends, not means. Writing in this vein, Waldron (2014) urges: “Deeper even than this is a prickly concern about dignity. What becomes of the self-respect we invest in our own willed actions, flawed and misguided though they often are, when so many of our choices are manipulated to promote what someone else sees (perhaps rightly) as our best interest? … I mean dignity in the sense of self-respect, an individual’s awareness of her own worth as a chooser.” An emphasis on the idea that people are making choices about *their* lives might reflect a commitment to respect for dignity and autonomy, not welfare at all.^7^ That form of respect might stand as a decisive objection to paternalism in policy and law.^8^

These arguments overlap with those in the canonical text on these questions, *On Liberty*, where Mill (2002) insisted:

The only purpose for which power can be rightfully exercised over any member of a civilized community, against his will, is to prevent harm to others. His own good, either physical or moral, is not a sufficient warrant. He cannot rightfully be compelled to do or forbear because it will be better for him to do so, because it will make him happier, because, in the opinion of others, to do so would be wise, or even right.

Mill offered a number of separate justifications for his famous Harm Principle, but his most emphatic, and the most relevant here, is epistemic. It is in the same family as the first of Bernheim’s: choosers are in the best position to know what is good for them. In Mill’s view, the problem with outsiders, including government officials, is that they lack the necessary information. Mill insists that the individual “is the person most interested in his own well-being,” and the “ordinary man or woman has means of knowledge immeasurably surpassing those that can be possessed by anyone else.” When society seeks to overrule the individual’s judgment, it does so on the basis of “general presumptions,” and these “may be altogether wrong, and even if right, are as likely as not to be misapplied to individual cases.” If the goal is to ensure that people’s lives go well, Mill concludes that the best solution is to allow people to find their own path.

That conclusion is echoed in Hayek’s (2013) suggestion that “the awareness of our irremediable ignorance of most of what is known to somebody [who is a chooser] is *the chief basis of the argument for liberty.*” For Hayek, the key contrast is between the chooser, who knows a great deal, and the outsiders, who show “irremediable ignorance,” especially if they are social planners.

Important strands in contemporary behavioral welfare economics make elaborate and instructive arguments in Hayek’s direction, suggesting that deference to individual choice is the right approach, subject to specific qualifications.^9^ Bernheim’s emphasis on “the central Cartesian principle that subjective experience is inherently private and not directly observable,” and on our unique knowledge of “how we feel,” fits well with Mill’s claim that the “ordinary man or woman has means of knowledge immeasurably surpassing those that can be possessed by anyone else.” When people choose chocolate over vanilla, salmon over tuna, basketball over football, rest over recreation, or spending over saving, they do so because they know what they like. Outsiders are most unlikely to have that knowledge.^10^

To be sure, we should be cautious here. Return to the cases of Masters, Lancaster, Harston, and Ullner. It is true that even if people usually have unique knowledge of “how they feel,” their knowledge on that count may not be perfect (Wilson, 2004). With respect to how choices will actually affect welfare, external observers might know far better, especially if the area requires technical expertise (Bubb & Pildes, 2014). Recall that choosers must solve a prediction problem; they must decide, at some point in advance of actual experience, about the effects of one or another option on experience (Kahneman *et al.*, 1997).^11^ To solve that problem, knowing “how they feel” is not enough. At a minimum, they must know “how they *will* feel,” and they might not know nearly enough to know that. In many cases, most dramatically that of a pandemic, the case for a mandate may well turn out to be plausible on welfare grounds; return to the case of Ullner.

But as a general rule, the claim for the epistemic advantages of choosers is more than plausible. At the same time, it has taken a real battering from behavioral findings.^12^

## 3. Direct and indirect judgments

Many people are troubled by that apparent battering.^13^ For current purposes, the most important challenge involves Premise 1. Behavioral findings seem to suggest that “people do not reliably exercise good judgment.” More precisely, behavioral science shows that people sometimes make mistakes about what would promote their own well-being, which means that in some cases, they are not the best judges of what would do that.^14^ If so, Mill’s epistemic argument is severely undermined. For purposes of law and policy, the door would seem open to paternalism, not only in the form of nudges, but also in the form of mandates and bans (Conly, 2013; Bubb & Pildes, 2014). At least this is so if welfare is our guide.

In what might plausibly be taken as an effort to reconstruct and even to rescue the essentials of standard welfare economics and Mill’s basic account, Bernheim (2016) responds that “the argument is faulty because it conflates what I will call direct and indirect judgments.” In this way, Bernheim is attempting to lay the foundations for choice-oriented methods for welfare analysis in the presence of behavioral biases. In his account, a direct judgment involves ultimate objectives, or outcomes that people care about for their own sake. An indirect judgment involves alternatives that lead to those outcomes. We might understand direct judgments to involve intrinsic goods, and indirect judgments to involve goods that are instrumental to their realization.

As an example, Bernheim points to a person, called Norma, who is choosing between two boxes, a red one containing a pear and a yellow one containing an apple. She prefers apples, but she chooses the red one, mistakenly ending up with a pear. This is a welfare-reducing indirect judgment, and Bernheim agrees that Norma has made a mistake. More broadly: “Behavioral economics and psychology provide us with ample reason to question certain types of indirect judgments.” In the face of a mistaken indirect judgment about money, safety, or health, a response might take the form of mandatory disclosure, or a warning, or another kind of nudge, such as a default rule; in extreme cases, it might justify a ban (Conly, 2013). But so long as we are speaking only of indirect judgments, we might insist that we have not departed radically from Mill’s general framework, amending it only to say that for indirect judgments, his epistemic argument sometimes fails. The cases of Marston, Lancaster, Harston, and Ullner can be understood in precisely these terms.

We can link Bernheim’s (2016) argument here with the suggestion that behavioral economics justifies “means paternalism” but not necessarily “ends paternalism” (Sunstein, 2014b). The basic idea is that people are sometimes mistaken about how to get to their own preferred destination. On that view, behaviorally informed interventions increase *navigability*, writ very large. A GPS device is a form of means paternalism. It allows drivers to specify where they want to go (and helps them to get there). A default rule, automatically enrolling people in some program, can be seen as means paternalistic insofar as it is thought that (most) people prefer to be enrolled in that program (but do not enroll, because they suffer from inertia) (Madrian & O’Shea, 2002). A calorie label can be seen as a form of means paternalism insofar as it improves people’s ability to make their own choices about how to promote their ends.

Many efforts to increase navigability, embodying a form of means paternalism, retain freedom of choice, and so can be seen as compatible with Mill’s Harm Principle (Sunstein, 2019a). But some do not. A ban on trans fats can be regarded as a form of means paternalism, at least if we are clear that the ban fits with, and does not undermine, people’s ends (Conly, 2013). Occupational safety requirements can be seen in similar terms, even if they override the choices of unrealistically optimistic workers who would be willing to run the risks that those requirements eliminate (Akerlof & Dickens, 1982). When behavioral research finds that people are making a mistake, we have a *behavioral market failure*, in the sense that people’s judgments lead to some kind of welfare loss, perhaps because of an identifiable behavioral bias (Bar-Gill, 2012; cf. Akerlof & Shiller, 2016). Much of behavioral law and economics is focused on that problem (Bar-Gill, 2012; Bubb & Pildes, 2014). As invoked for purposes of law and policy, behavioral market failures typically involve means paternalism (Bar-Gill, 2012). To see the underlying issues, let us use Bernheim’s terminology, distinguishing between direct and indirect judgments.

Does that distinction work? How helpful is it? Insofar as we are dealing with unambiguously indirect judgments, and thus respecting people’s ends, the problem of unjustified paternalism seems to be solved. But when, in ordinary practice, are those involved in law or policy dealing with direct judgments? An initial problem is that if Norma prefers apples to pears, it is natural to ask whether an indirect judgment is involved as well. Apples are good, but it would be hard to justify the conclusion that they are “ultimate objectives, or outcomes that people care about for their own sake.” (Friendship might be an intrinsic good; not so much apples.) If Norma prefers apples to pears, it is probably because she thinks that they taste better or perhaps that they are healthier. But perhaps she is wrong on either or both those counts. In other words, her preference for apples is itself an indirect judgment, and she might be badly mistaken, perhaps because of a behavioral bias.

The legal applications are numerous, including regulatory responses to unhealthy eating (Rabin, 2013), insufficient savings (Bubb & Pildes, 2014), and “dark patterns” online (Ligori & Strahilevitz, 2019). Bernheim (2016) is alert to this point and adds:

Now let us add a wrinkle: assume Norma’s ultimate goal is to achieve certain mental states (“internal goods”). From that perspective, all consumption items (“external goods”) are means to ends, and choices among them always involve indirect judgments. Moreover, just as Norma may misjudge the contents of a box, she may also misapprehend the relationships between consumption goods and mental states. However, assuming she is sufficiently familiar with apples, pears, and bananas to understand the consequences of eating each, her indirect judgments among open boxes will be correctly informed, and hence will faithfully reflect her direct judgments.

What is rightly added here is more than a “wrinkle.” It is fundamental to behaviorally informed law and policy, and also to behavioral welfare economics. Norma is probably not concerned only with internal mental states (she might well care about price, health, and morality), and as we shall see, this is a significant point, counting against hedonic accounts of welfare;. But when it comes to food choices, her mental state is almost certainly something that she cares about. Consumption choices are typically means to ends, and in that sense they typically involve indirect judgments. Moreover, the assumption of sufficient familiarity might turn out not to hold. For many choices, people are not sufficiently familiar with the options “to understand the consequences” of each, and even if they are, they might suffer from some kind of behavioral bias, such as present bias.^15^ In this light, we should add that for Norma’s choice of apples to be a good measure of her welfare, she must not only be informed (“sufficiently familiar”), but also free from any such bias. Modifying Mill, we might adopt this working presumption in favor of an amended version of Premise 1, designed to orient behavioral welfare economics as applied to law and policy:

*Working presumption: Each of us should be taken by outsiders to be the best judge of what will promote our own well-being, to the extent that we are adequately informed and sufficiently free of behavioral biases.*

The working presumption is an effort to build on choice-oriented methods for welfare analysis, as in Bernheim’s approach, but for two reasons, it is more cautious. First, it is only a presumption. Second, it does not depend in any way on a distinction between direct and indirect judgments, or ends and means, though it is true that in application, it is usually likely to support interventions in the interest of means paternalism. Note also the use of the words “should be taken by outsiders to be,” as distinguished from the more dogmatic “are”; I return to the difference below.

Of course, there are questions about how to operationalize the working presumption. If we care about welfare, it would make sense to examine what choices people make when they are actually well-informed; to see what choices people make when they do not suffer from limited attention and are in a position to evaluate all relevant facets of an option; to use people’s active choices rather than passive ones, which may be a product of inertia; and to use otherwise unbiased choices, such as long-run choices based on a realistic understanding of facts, rather than biased ones, such as those that reflect present bias or optimistic bias (Allcott & Sunstein, 2015). Ideas of this kind can be seen as an effort to draw on a broadly Millian understanding, respectful of private choices, while also recognizing and giving weight to information deficits and behavioral biases.^16^ They might provide a way to discipline behavioral welfare economics in areas that include savings behavior, decisions with respect to energy-efficient products, and choices of high-calorie or low-calorie food (Thunstrom, 2019).

By contrast, one of Bernheim’s goals is to insist on respect for direct judgments. Instead of the working presumption immediately above, he argues in favor of two premises (Bernheim & Taubinsky, 2018):

*Premise A: With respect to matters involving either direct judgment or correctly informed indirect judgment, each of us is the best arbiter of our own well-being.*

*Premise B: When we choose, we seek to benefit ourselves by selecting the alternative that, in our judgment, is most conducive to our well-being.*

In defense of Premise A, Bernheim (2016) urges that existing economic research does not “provide evidence that people exercise poor direct judgment—for example, that they like certain goods or experiences ‘too much’ and others ‘not enough.’” On the contrary, he maintains, “[t]he occasional objection to a direct judgment entails nothing more than a difference of opinion between the analyst and the consumer as to what constitutes a good or fulfilling life.” The conclusion might be right, but these are strong words—too strong, in my view, and for three separate reasons. First, they disregard the possibility that people are genuinely making a mistake about what makes human lives go well—not because of a mere difference of opinion between the analyst and the consumer, but because that conclusion follows from *any* reasonable judgment about what it means for a human life to go well. Second, they take a stand on some contentious philosophical issues about welfare (a point taken up below). Third, they collapse the distinction between two very different questions: (*a*) Do people know what they will like, before they have it? and (*b*) Do people know what they like, when they are having it? Even if we think the answer to the second question is usually “yes,” we have not answered the first question.

Repeating the basic claim elsewhere, Bernheim and Taubinsky add: “Thus there is no objective foundation for overturning the presumption in favor of a direct judgment and declaring the analyst’s perspective superior” (Bernheim & Taubinsky, 2018). But the underlying issues are more complicated than that. It is true that if all we have is a “difference of opinion” between the analyst and the chooser, we do not, by stipulation, have an “objective foundation” for favoring the views of the analyst. But what if the analyst has actual data, suggesting that people’s direct judgments produce large welfare losses (Levitt, 2016)? What if the analyst has information about what people are likely to like, and what if that information suggests that people’s ex ante predictions are incorrect? What if the analyst has an account of what makes for a good or fulfilling life, and not simply an “opinion” (Feldman, 2010)?

## 4. Practice and theory

I now offer three claims. The first is that for behavioral economics and behaviorally informed law and policy, indirect judgments, or judgments about means, really are the coin of the realm. If we are speaking about inertia, present bias, unrealistic optimism, probability neglect (Sunstein, 2002), or limited attention, we are almost always dealing with judgments or decisions that might defeat people’s own ends. And if we are speaking of default rules, disclosure, reminders, warnings, or uses of social norms, we are almost always dealing with efforts to encourage people to choose better means to achieve their own ends. Behavioral welfare economics typically deals with indirect judgments, and if it embraces paternalism, it is means paternalism.

The second is that with respect to direct judgments, behavioral welfare economics, like standard welfare economics, should proceed with humility. One reason is empirical; another is normative. To say that informed choosers are the best arbiters of their own welfare is to take a contested stand on how to think about the very idea of welfare. In the liberal tradition, time-honored ideas about autonomy and welfare do support a working presumption in favor of something like that proposition. But the presumption should be embraced with caution, and with an understanding of what kind of stand it is taking.

The third claim, and the most ambitious, involves the proper understanding of welfare. Put too simply, the claim is that each of the three prevailing theories in philosophical circles—rooted in preferences, subjective well-being, and objective goods (Adler, 2011)—runs into serious problems. People’s preferences, understood as their ex ante judgments about what to choose, will not always promote their welfare, simply because they might be inadequately informed or suffer from some kind of behavioral bias.

At the same time, we should not understand welfare in purely hedonic terms. People might choose to have a more meaningful life even if they end up sadder or more anxious. They might care most about meaningfulness, not happiness or a lack of anxiety. People might choose a life that they consider to be better, even if they are more distressed as a result of choosing that life. These points might be taken to lead us to objective-good theories of well-being, and across a certain domain, those theories have considerable appeal. At the same time, what is good for John might not be good for Jane, and objective-good theories struggle (I suggest) to take account of heterogeneity.

The working presumption, sketched above, is not meant to take a stand on the deepest philosophical questions; my hope is that it can be the outcome of an incompletely theorized agreement. But I shall try to bring the working presumption in contact with those questions, and to show its appeal on pragmatic grounds. To simplify a complex story: The working presumption embodies an understanding that preference-based accounts of welfare must recognize that people might suffer from insufficient information and behavioral biases. It offers a highly qualified version of those accounts. The working presumption also recognizes that purely hedonic accounts of welfare, or those that focus on subjective well-being, miss the fact that reasonable people reasonably care about things other than their moods, their subjective well-being, or even their experience. Recall that they might, for example, want to live meaningful lives. The working presumption does not embrace objective-good accounts of welfare,^17^ on the ground that reasonable people choose to live a great diversity of good lives; but it leaves open the possibility that some lives are objectively bad.

### 4.1. The pervasiveness of indirect judgments

As applied to law and policy (Thaler & Sunstein, 2009; Conly, 2013; Mullainathan & Shafir, 2013),^18^ almost all of behavioral economics involves indirect judgments. The bulk of behavioral research involves such judgments as well (Kling *et al.*, 2012). For example, the choice of one product over another—say, the Toyota RAV4 over the Toyota RAV4 Hybrid—will almost certainly be a result of a number of subsidiary judgments: how the preferred vehicle looks, how it drives, how reliable it will be, how big it is, the storage space, the purchase price, the anticipated cost of operation, and how often one will have to go to a gas station and refuel. What is the direct judgment here? We should agree that if a consumer named Susan makes some kind of clear mistake—she wants a smaller car and wrongly believes that the hybrid model is bigger—she is like Norma, selecting the wrong box. But suppose that Susan makes a subtler error: She is insufficiently responsive to fuel savings, in the sense that she would have saved a lot of money with the RAV4 Hybrid, but that because of present bias, she decided against it (Gillingham *et al.*, 2019). Unless we introduce other considerations (such as a liquidity constraint), Susan was mistaken. She was not, in this case, the best judge of her own well-being. Some kind of intervention would appear to be necessary, perhaps in the form of clear disclosure of relevant information (if the disclosure works, as it might not, to overcome present bias).

Perhaps we should say that Susan was not “sufficiently familiar with” the two kinds of cars “to understand the consequences” of choosing one or the other, and so her indirect judgment between the two was not “correctly informed, and hence” did not “faithfully reflect her direct judgments.” Fair enough. But if that is the case, exactly how much remains of the idea of deference to individual judgment in cases of genuine or realistic interest to law and public policy^19^? A great deal still, perhaps, but a lot less than suggested by standard economic theory. In many circumstances, people are good indirect choosers, likely better than anyone else, but when they lack information or suffer from a behavioral bias, their indirect choices will not be reliable. Whether particular choices fall in that category is an empirical question.

To broaden the viewscreen: Many laws and regulations involves externalities, and for that reason, much of the welfare analysis need not draw on behavioral economics; it is conventional. For example, fuel economy regulations impose costs, which can be calculated (and which are imposed largely on consumers, in the form of higher sticker prices) (Bento *et al.*, 2019). Those regulations also reduce air pollution, including greenhouse gas emissions; monetization is more challenging here, but standard tools are available to do exactly that (Greenstone, 2013). To this extent, the welfare analysis should be familiar (Gayer & Viscusi, 2013). The distinctly behavioral dimension comes from possible errors on the part of consumers, who may not be giving sufficient weight to economic savings (from reduced gas usage) and also to time savings (from fewer visits to the gas station) (see the catalogue in ibid.). For behavioral reasons, consumers may indeed be making errors, which means that they stand to gain a great deal from fuel economy mandates, even when those mandates override their choices (for a skeptical view, see ibid.; for a less skeptical view, see Allcott & Sunstein, 2015).

Consumers can, of course, choose fuel-efficient vehicles if they like. If they do not, perhaps it is because the more fuel-efficient vehicles are smaller or less powerful, or because they are inferior along some other dimension. The behavioral question is whether consumers neglect fuel economy because of (for example) present bias, myopic loss aversion, or limited attention, or instead consider fuel economy but find it outweighed by other factors. The behavioral hunch is that present bias or limited attention do play a role, but a hunch is not evidence.^20^ Behavioral welfare economics would carefully investigate the hunch, and consider consumer savings to the extent that the evidence suggests that they are real.^21^ In fact that is a central, even defining question in contemporary regulatory policy, bearing on energy efficiency requirements as well as fuel-economy regulation (Gayer & Viscusi, 2013; Allcott & Sunstein, 2015; Allcott & Knittel, 2019).

### 4.2. Defining direct judgments

What about people’s direct judgments? Do they deserve deference? When? To make progress on these questions, we have to know how to identify them, which means that we have to solve what might be called the level of abstraction problem. Return to the case of Norma. We could say that she prefers Yellow Box to Red Box, believing that Yellow Box contains apples; that is clearly an indirect judgment, because she wants an apple, not a pear. But we could also say that what she wants is a good snack rather than a less good snack (bracketing the question of what, exactly, makes a snack good), and that the choice of an apple is an indirect judgment. Or we could say that what she wants is a good afternoon, or day, or year, or life, and the choice of a good snack is instrumental to one of those things.

Suppose that direct judgments are described at the highest level of abstraction—say, having a good life (without specifying what that means, and on the assumption that a good life, properly defined, is intrinsically good). If so, essentially all real-world judgments are indirect judgments, in the sense that they are meant as ways of getting a good life. The problem is that if direct judgments are described at the highest levels of abstraction, and if the claim is that we must respect such judgments, outsiders (including regulators) are not much constrained even if they accept that claim. The reason is that outsiders (including regulators) are always, or almost always, dealing with indirect judgments, so long as direct judgments are taken at the highest levels of abstraction.

It would be possible to understand direct judgments at a lower level of abstraction. Choosers make judgments about what kind of day they want to have or what kind of life they want to lead. They might prefer high-calorie, full-sugar soda to diet drinks, or pizza to salad, even if they gain weight. They might like basketball but not football. They might want to devote themselves to family. They might want to devote themselves to some cause. They might want to pursue art or sport. They might want to marry, or not. They might want to get drunk a fair bit, or not. They might want to live exciting, risk-filled lives, even at some cost to their health and longevity. Or they might want to live stable, risk-free lives, if that is the way of increasing the number of years they have on the planet. Which of the resulting judgments is direct, and which is indirect? More fundamentally: Does anything in behavioral economics demonstrate that people are mistaken with respect to these judgments?

These are not the easiest questions to answer. It is one thing to insist that people choose inferior health care plans, given their situations (Gillingham *et al.*, 2019), that they make some bad food choices (Rabin, 2013), given their overall concerns, or that they do not purchase fuel-efficient motor vehicles when it would be in their economic interest to do so (Gillingham *et al.*, 2019). It is quite another to insist that they choose the wrong sorts of days or lives. Do behavioral economists have anything to say on that question? If the answer is “yes,” it might be for empirical reasons. A body of research in behavioral science points to “hedonic forecasting errors,” which occur when people make mistaken predictions about the effects of outcomes or options on their subjective well-being (Gilbert *et al.*, 1998; Gilbert & Wilson, 2000).

Because the idea of “hedonic forecasting” is a bit narrow, we might ask whether people make “welfare forecasting errors,” that is, whether they make mistaken judgments about what will increase their welfare. They might think that their lives will be better if they marry, but they might be wrong. If so, we might be tempted to say that their judgment was indirect, which would bring us back to the level of abstraction problem. And if they are making the wrong choices, it probably must, simply as a logical matter, be because of a lack of information or some behavioral bias, though we might not be able to identify it, and though it might not be part of the standard catalogue of behavioral biases (see the long catalogue in Pohl, 2016).

### 4.3. Welfare

The largest question, of course, is how to define welfare. As noted, the philosophical literature distinguishes among three different theories: preference-based theories, hedonic theories, and objective-good theories (Adler, 2011). Economists are drawn to the former, but if welfare is what matters, and if respect for preferences leads to welfare losses, such theories have rserious problems. If those problems lead us to embrace hedonic theories, emphasizing subjective well-being, we can make real progress in understanding the idea of forecasting errors. People might think that they would be happier in California and therefore move there, but perhaps they would not be happier at all (Schkade & Kahneman, 1998).^22^ People might think that they would be unhappier if they left their current personal situation, but they might be quite mistaken on that point (Levitt, 2016).^23^

Should we embrace hedonic theories? Many people think so. But such theories have serious limitations. I have already signaled some of them, but to be more concrete, consider some cases:

(i) John is deeply committed to public service; he wants to make the world better. He works long hours, and he does not especially enjoy doing that. His days are not a great deal of fun. But he does not want to do anything else.
(ii) Mary is a professional tennis player. She has been playing for most of her life. To her, tennis is not much fun. But she wants to pursue excellence. She aims to see how good she can get.
(iii) Frances is a lawyer. She is excellent at her job. She is an intense person. She likes winning; she finds it rewarding. But she also finds it stressful. She is not particularly happy.

These cases reflect what should be an obvious fact, which is that people care about things other than their hedonic state. To be sure, we could understand subjective well-being to include an array of values that go beyond hedonics; a sense of meaning, goodness, devotion, or excellence could be included in the catalogue of what people care about. But if we do that, it is not clear that we are really speaking of *the quality of people’s experiences*, which is often what is meant by subjective well-being. People care about things other than that quality. They might care about meaningfulness or excellence for their own sake, and not because they affect the quality of their experiences. They might choose options that do not improve their experiences, but that make for a worthier or more meaningful life. (Of course, it is true that a worthy life, or a meaningful life, might also have beneficial effects on people’s experiences.)

It is for the reason that many people, including many economists, are drawn to preference-based theories, which can claim to take on board everything that people value, whether or not their experiences are improved. We have seen that preferences can lead to bad choices, in terms of what people care about, in the face of a lack of information or a behavioral bias. But we can take that to be a friendly enough amendment to preference-based accounts. After all, it gives authority to (suitably purified) preferences. As we have seen, one problem is that preference-based accounts pay too little attention to behavioral biases; perhaps that problem can be handled with suitable purification. But there is another problem, which is the risk that such accounts might pay too little attention to the ingredients of what is, for essentially all people, a good human life. If so, we might be drawn to objective-good accounts.

Such accounts take many forms. All of them would question the view that people’s preferences deserve the kind of authority that many people, including many economists, would give them. Many philosophers embrace what they call “perfectionism,” urging that some kinds of lives are simply better than others (Hurka, 1996).^24^ Some liberals are perfectionists; they give pride of place to a distinctive understanding of freedom (Raz, 1985). Some perfectionists are Aristotelian, emphasizing Aristotle’s notion of functioning and an understanding of what it means for a human being to be truly human (Foot, 2001).^25^ Marx was a perfectionist (Elster, 1985). Some forms of perfectionism have religious foundations; consider Aquinas.

On grounds of either autonomy (invoking Kant) or welfare (invoking Mill), we might want to reject perfectionism (Conly, 2013). But to say the least, these are complicated normative questions, and nothing in standard economics, or behavioral economics, is equipped to answer them. It does seem reasonable to say that if preferences are an adaptation to injustice or acute deprivation, they do not deserve authority (Elster, 1983). It also seems reasonable to say that if people’s preferences lead them to have objectively terrible lives, something has gone wrong. Objectively terrible lives might be painful, brutal, or short. Perhaps we can say that in such cases, people are almost certainly suffering from a lack of information or from a behavioral bias. But perhaps not; perhaps they prefer something that makes their lives painful, brutal, and short. It is in such cases that objective-good accounts have force.

This is not the place to try to answer some of the deepest questions in political philosophy. My main goal is to identify them. As John Rawls wrote in an unpublished manuscript, “We post a signpost. No deep thinking here; things are bad enough already.” It makes pragmatic sense to say that across a wide range of choices, people’s informed and unbiased preferences generally deserve authority, because they are the best available reflection of what people actually care about. An important qualification arises when people’s preferences lead them in directions that make their lives go less well (by their own lights). If they care about their own welfare, and if their choices compromise their welfare, there is a problem. Another qualification arises when their preferences lead them in the direction of what is, by any reasonable account, an objectively bad life. Because the range of objectively good or not-bad lives is (in my view) very wide, we should much hesitate before bringing that qualification into play. All this is enough, I hope, to support an incompletely theorized agreement on the working presumption, in the form of a willingness to embrace it even if we are puzzled or disagree about the largest questions.

## 5. Paths forward

In some cases, people lack information (Bar-Gill, 2012). In other cases, we can identify a behavioral market failure, in the sense that people fall prey to an identifiable behavioral bias, and their choices make their lives go worse by their own lights (Bubb & Pildes, 2014). When this is so, some kind of corrective response is likely to be a good idea, perhaps in the form of a nudge, perhaps in the form of a tax, perhaps in the form of a mandate. One more time: In a free society, and notwithstanding the philosophical concerns, it nonetheless makes pragmatic sense for those involved in law and policy to adopt a working presumption: *Each of us should be taken by outsiders to be the best judge of what will promote our own well-being*, *to the extent that we are adequately informed and sufficiently free of behavioral biases.* In practice, that presumption can be disciplined by asking four subsidiary questions:

*(1) What do informed choosers choose?*

*(2) What do active choosers choose?*

*(3) In circumstances in which people are free of (say) present bias or unrealistic optimism, what do they choose?*

*(4) What do people choose when their viewscreen is broad, and they do not suffer from limited attention?*

Some of these subsidiary questions can be answered empirically. Consider, for example, the question whether and to what extent an absence of information leads consumers to fail to choose fuel-efficient motor vehicles. Experiments might be designed to provide consumers with relevant information and see what they choose (Allcott & Knittel, 2019). The choices of informed consumers might be taken as the foundation for analysis. If most consumers make an active choice to enroll in overdraft protection programs under an opt-in regime, there is at least some reason to think that such programs are in their interests (Sarin, 2019).^26^ (I am bracketing the potential benefits of targeted or personalized programs.) Experiments might also be designed to make the potential economic savings of (say) energy-efficient lightbulbs highly salient, at least potentially overcoming present bias and limited attention (Allcott & Taubinsky, 2015). If consumers choose or do not choose such lightbulbs in such circumstances, we will have learned something about what is likely to increase their welfare.

In principle, efforts to answer these subsidiary questions should help with cost–benefit analysis, where it is often challenging to know how to proceed when behavioral findings seem to cast doubt on standard uses of revealed preferences.^27^ Answers to the subsidiary questions might also allow considerable room for regulatory interference with indirect judgments; such answers might well authorize means paternalism, often in the interest of increasing navigability.

With respect to people’s ends, operating at a high level of abstraction, those who offer the working presumption insist on considerable deference to freedom of choice. But they recognize that the underlying justifications for the presumption, founded on ideas about autonomy and welfare, cannot avoid taking some kind of philosophical stand. They provide those justifications with conviction, but also with humility. Recognizing that large questions can be found in the background and sometimes the foreground, they hope to achieve an incompletely theorized agreement on behalf of the presumption: an agreement among those who are uncertain about the most fundamental issues, or who disagree intensely about how to resolve them.

## Footnotes

1 The presumption could be fortified with reference to the potentially self-interested or malevolent incentives of those who seek to interfere with people’s judgments. I am bracketing that important point here.

2 For valuable discussion, see Goldin (2015, 2017). To summarize a complex argument, Goldin argues for a distinction between inconsistent choosers, whose choice depends on the default, and consistent choosers, whose choice is impervious to the default. In his account, it is “quasi-paternalistic” to nudge inconsistent choosers. That is a helpful distinction. At the same time, consistent choosers might be impairing their own welfare, perhaps because of present bias or unrealistic optimism. On the philosophical foundations, see Pettigrew (2020).

3 For different efforts to meet those challenges, see Thaler and Sunstein (2009) and Goldin (2015).

4 For valuable discussion of foundational issues, see Adler (2011). On law and policy, see Bronsteen *et al.* (2013).

5 For one example in a specific context, see Gayer and Viscusi (2013). For a general account, see Wright and Ginsburg (2015).

6 For a series of objections, see Conly (2013).

7 Some questions about this idea are raised in Sunstein (2014a).

8 For a defense of coercive paternalism, see Bubb and Pildes (2014). By design, Bubb and Pildes do not engage this kind of normative objection: “We thus put aside various external critiques that could be, and have been, mounted. Autonomy- or liberty-based political theories argue, for example, that these values should have priority over welfare maximization.” The only point is that if a Kantian idea, of the sort invoked by Waldron (2014) and Bernheim (2016), is in play, it would serve as a check on imaginable claims in behaviorally informed law and policy.

9 Bernheim and Taubinsky (2018) can easily be read in this way. For an influential early effort, see Glaeser (2006).

10 A rejoinder can be found in Conly (2013): “We don’t regard it as insulting to assume that the man on the street can’t do quantum mechanics, because he can’t (unless you’re on a very special street). The paternalist believes that it is the facts that suggest a change in the status we accord people; change from what we might have thought about ourselves to a more realistic acceptance of our inabilities. The suggestion here is simply that we should treat people in accordance with their real abilities and their real limitations.”

11 Sunstein (2019b) finds what seems to be a significant mistake, on the part of consumers, with respect to a technology designed to make driving safer.

12 Ambitious accounts are Conly (2013) and Bubb and Pildes (2014).

13 For different perspectives, see Allcott and Sunstein (2015), Goldin (2017), and Bernheim and Taubinsky (2018).

14 For some vivid examples, see Abaluck and Gruber (2009, 2013), Afendulis *et al.* (2015), and Bhargava et al. (2015, 2017).

15 Cigarette smoking is an example (Masiero *et al.*, 2015).

16 Akerlof and Shiller (2016) is in the same vein, though it emphasizes the active efforts by sellers to exploit those deficits and biases, or to “phish” for them.

17 It is important to say here that objective-good accounts take different forms and they might well recognize that many different kinds of lives can be objectively good.

18 We can understand policy to include some behaviorally informed judgments of private institutions (Laibson, 2018).

19 Recall Bernheim’s acknowledgment of the possibility that “all consumption items ‘external goods’ are means to ends, and choices among them always involve indirect judgments.” It is true that some governments impose coercive controls on intimate aspects of people’s lives—as, for example, by criminalizing same-sex relations—and these controls might, at some point, be defended on behavioral grounds. But in the institutions that are now using behavioral economics, indirect judgments are the lay of the land, and the same is true of academic research that finds mistakes or recommends a behaviorally informed intervention.

20 The hunch is questioned in Gayer and Viscusi (2013) and in Allcott and Knittel (2019); it is supported in Gillingham *et al.* (2019).

21 The issue remains debated, and in my view, unresolved (Gayer & Viscusi, 2013; Allcott & Knittel, 2019; Gillingham *et al.*, 2019).

22 Bernheim and Taubinsky (2018) raise a number of objections to reliance on subjective well-being. As noted, it is right to insist that subjective well-being is not the only thing that people care about; for example, choosers might sacrifice their subjective well-being for the sake of others, for the sake of living meaningful lives, or for the sake of moral goals. But the case for relying on choices, rather than subjective well-being, is not made out by that point. In some contexts, choices really are an effort to promote subjective well-being, and they go wrong.

23 In general, Levitt (2016) finds excessive caution in making life-changing decisions.

24 For a defense of liberal perfectionism, see Raz (1985); for critiques of perfectionism, see Rawls (1991) and Conly (2013). For a general account, see Zalta (2017).

25 Nussbaum (2000) can be placed in the same extended family. For an especially clear treatment, see Nussbaum (1993).

26 It is important to be careful here; active choices might be uninformed or behaviorally biased.

27 One example is the continuing dispute over the benefits, to consumers, of fuel economy and energy efficiency requirements (Allcott & Taubinsky, 2015). Another example is the dispute, also continuing, about how to value reductions in smoking; to what extent do they improve the welfare of former smokers? (Levy *et al.*, 2018). The framework introduced in Levy *et al.* (2018) is highly compatible with the analysis here.
